# Clinical Outcomes of Keratolimbal Allograft Transplantation With Oral Mucosal Transplantation for Limbal Stem Cell Deficiency With Symblepharon

**DOI:** 10.1155/sci/8426444

**Published:** 2025-09-24

**Authors:** Shang Li, Yinghui Wang, Zhiqun Wang, Shijing Deng, Lan Lv, Ying Jie

**Affiliations:** Beijing Institute of Ophthalmology, Beijing Tongren Eye Center, Beijing Tongren Hospital, Beijing Tongren Hospital, Capital Medical University, Beijing Ophthalmology and Visual Sciences Key Laboratory, Beijing 100730, China

**Keywords:** keratolimbal allograft, limbal stem cell deficiency, ocular surface reconstruction, oral mucosal transplantation, symblepharon

## Abstract

**Objective:** To report preliminary clinical outcomes of keratolimbal allograft (KLAL) transplantation with oral mucosal transplantation (OMT) for the treatment of limbal stem cell deficiency (LSCD) with symblepharon.

**Methods:** This is a retrospective review of patients with LSCD and symblepharon who underwent KLAL transplantation with OMT at the Department of Ophthalmology, the Capital Medical University of Beijing Tongren Hospital between 2022 and 2024. Patients with at least 3 months of postoperative follow-up and adequate pre or postoperative records were enrolled. Grades of symblepharon, corneal conjunctivalisation, vascularization, opacification, fornix depth, and best corrected visual acuity (BCVA) were evaluated preoperatively and postoperatively. In five cases, in vivo confocal microscopy (IVCM), impression cytology (IC), and immunofluorescence (IF) were performed.

**Results:** At a postoperative follow-up of 8.27 ± 5.80 months (3–22 months), 10 of 11 eyes (90.91%) maintained a successful outcome. The grades of symblepharon, corneal conjunctivalisation, vascularization, and opacification were significantly improved after surgery (*p* ≤ 0.01). Significant deepening of the fornix depth in the superior (*p* ≤ 0.01) and inferior conjunctival (*p* ≤ 0.05). Two-line improvement in BCVA was seen in 8 eyes (72.73%). Recurrence of LSCD occurred in 1 eye (9.09%). Morphology and structure of corneal epithelial cells and epithelial transition around the KLAL segments were detected by IVCM, IC, and IF.

**Conclusions:** From the preliminary clinical results, KLAL with OMT is a safe and effective surgical technique for LSCD with symblepharon, maintaining epithelial stability, and restoring the patient's ocular anatomy.

## 1. Background

Corneal limbal stem cells (LSCs) are located at the basal epithelium of the limbus and are important in maintaining corneal epithelial homeostasis. Damage to the limbus, such as chemical or thermal burns, Stevens–Johnson syndrome (SJS), infectious diseases, or surgical injuries, can lead to LSC deficiency (LSCD) [1]. Clinical features of the disease include corneal conjunctivalization, neovascularization, recurrent or persistent epithelial defects, and symblepharon [2]. For LSCD with symblepharon, the typical order for surgical intervention is correction of eyelid abnormalities first, for example, injection of Mitomycin-C, conjunctival autograft transplantation, amniotic membrane transplantation (AMT), and so on, followed by ocular surface reconstruction [3]. When symblepharon is extensive and cannot be repaired by the amniotic membrane or conjunctiva, oral mucosal transplantation (OMT) can be effective [4].

Depending on the extent of corneal and limbal involvement detected by clinical examination, LSCD can be divided into three stages as shown in Table 1 [5]. In patients with stage II and III LSCD, autograft transplantation harvested from the contralateral healthy eye can increase the risk of LSCD in the donor eye due to the large excision of the limbus. Keratolimbal allograft (KLAL) from cadaveric donors is an option for restoration of the ocular surface in bilateral total LSCD; unilateral extensive LSCD; and refusing surgery on the contralateral healthy eye [6]. Traditionally, KLAL is a crescent-shaped lamellar graft that includes the smallest portion of scleral tissue, the entire limbus, the cornea's outermost layer, and more LSCs [7]. For severe LSCD, 2–3 crescent-shaped implants must be pieced together. Previous studies have shown that the long-term success rate of KLAL transplantation ranges from 27% to 72% [6]. The main complications leading to surgical failure include immune rejection and recurrent symblepharon [8, 9].

To improve LSCD and symblepharon simultaneously, reconstruct the conjunctival sac, and prevent the recurrence of symblepharon, we combined KLAL transplantation with OMT. For total LSCD, we innovatively used ring-shaped KLAL. In this study, we retrospectively report the surgical outcomes of 11 eyes of 10 patients diagnosed with partial or total LSCD and received the procedure.

## 2. Methods

### 2.1. Study Design and Patients

This was a single-center, retrospective study. The protocol of this study was consistent with the principles of the Declaration of Helsinki. This study was approved by the Ethics Committee of Beijing Tongren Hospital, Capital Medical University (TRECKY2020-004). Surgery was performed on patients who had recurred LSCD with symblepharon after AMT or medical treatment. The procedure was performed 3 months after the last medical operation. Medical records of 11 eyes of 10 patients who were clinically diagnosed with LSCD underwent KLAL transplantation with OMT from January 2022 to April 2024 were reviewed.

### 2.2. Inclusion and Exclusion Criteria

Cases who underwent KLAL transplantation with OMT due to chemical or thermal burns and were followed up for more than 3 months were included. Patients with glaucoma, diabetes, and autoimmune diseases were excluded. Patients who had received OMT, corneal LSC transplantation, or penetrating keratoplasty before this surgery were excluded.

### 2.3. Data Collection

Data were collected on every visit, and the completed form was filed in the medical record. The patient's demographic and clinical data included age, gender, etiology and date of injury, details of prior surgery, best corrected visual acuity (BCVA), intraocular pressure (IOP), details of intraoperative surgery, postoperative complications, duration of follow-up, and status of the ocular surface at each visit were recorded (Tables 2 and 3).

### 2.4. Surgical Technique

All surgeries were performed jointly by Dr. Shang Li and Dr. Lan Lv. The graft was stored uniformly by the eye bank of our hospital. It is stored individually in Optisol preservation solution for 5–9 days with a temperature of 2–8°C.

**Figure 1:**
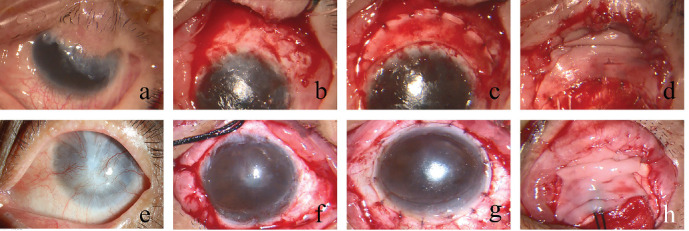
Surgical technique. (a, e): Appearance of the ocular surface before surgery. (b, f): The scar tissue of the ocular surface was removed to expose the implant bed. (c, g): Lamellar thinning of the KLAL segments. (d, h): Oral mucosal graft was secured to the defects of conjunctiva.

Briefly, a limbal peritomy was performed according to the extent of limbal involvement. Abnormal fibrovascular pannus, epithelium, and subconjunctival fibrovascular tissue were removed from the corneal surface to expose the sclera and corneal stroma (Figure 1b and f). The posterior stroma, Descemet's membrane, and endothelium of the donor limbus were removed by lamellar dissection with a sharp rounded crescent blade and small curved vascular clamp. Crescent-shaped implants were used for partial LSCD (Figure 1c), and ring-shaped implants were used for total LSCD (Figure 1g). Crescents were placed around the recipient cornea with the anterior corneal edge overlying the recipient limbus and secured with 10-0 nylon sutures (Figure 1d). An oral mucosal graft (OMG) of 15 × 10 mm was harvested from the patient's lower lip. The graft was sufficiently thinned by removing all submucosal tissue, fat, and glands. OMG was transferred to the defects of the conjunctiva and fixated with 8-0 nylon sutures to reconstruct the bulbar conjunctiva, the conjunctival sac, and palpebral conjunctiva (Figure 1d,h).

### 2.5. Postoperative Management and Follow-up

Postoperatively, chlorhexidine mouthwash was prescribed for 2 weeks. Recipient eyes were prescribed 1% prednisolone acetate ophthalmic suspension eye drops (Allergan Pharmaceuticals Ireland, Dublin, Ireland) four times/day for 1 month; gatifloxacin eye ointment (Xingqi Eye Medicine Co., Ltd., Shenyang, China) and recombinant bovine basic fibroblast growth factor eye drops (Yisheng Biopharmaceutical Co., Ltd, Zhuhai, China) four times/day for 1 month. 0.1% tacrolimus eye drops (Senju Pharmaceutical Co., Ltd., Japan) four times/day for 6 months. Patients were followed for at least 3 months. At every visit, BCVA was measured, and a slit lamp examination was undertaken. In vivo confocal microscopy (IVCM), impression cytology (IC), and immunofluorescence (IF) were performed in five cases to confirm the epithelial phenotype postoperatively.

### 2.6. IVCM

IVCM was performed using Heidelberg Retina Tomograph (HRT3; Heidelberg Engineering GmbH, Heidelberg, Germany). Before examination, the recipient eye needed to be topically anesthetized by proparacaine hydrochloride eye drops (Alcon-Couvreur, Bornem, Belgium). A drop of Carbomer gel was applied to the disposable cap and the cornea to protect the ocular surface. TomoCap was then installed on the corneal module and advanced gently to touch the cornea. Shoot the center of the cornea, limbus, and limbal tissues, and take three or more images at each location [10].

### 2.7. IC

IC was performed using multiple square biopore membranes. The diameter of the membranes was 5 mm. Under topical anesthesia, the membranes were placed on the corneal surface for 5 s. The membranes were soaked in formalin to fix the color and then stained with periodic acid–Schiff (PAS). After that, photographs were taken with a microscope.

### 2.8. IF

Multiple square biopore membranes were placed over the limbus of the recipient's eye for 5–10 s. The membranes were fixed in acetone at −20°C for 1 min and peeled off from the plastic frame. The cells were permeabilised with 0.2% Triton X-100 (T8787, Sigma-Aldrich, St. Louis, MO, USA) in phosphate buffered saline (PBS) for 20 min at room temperature. They then were treated with 2.5% bovine serum albumin (cat number, Sigma-Aldrich) in PBS for 20 min at room temperature to block nonspecific reactions and subsequently incubated in 1:100 rabbit antihuman MUC5AC antibody (ml086329, Shanghai Enzyme-linked Biotechnology Co., Ltd., China), 1:100 mouse antihuman CK7 antibody (MA1-06315, Abcam, Cambridge, UK), and 1:400 rabbit antihuman P63 polyclonal antibody (12,143-1-AP, Proteintech Group, Inc., USA) for 1 h at room temperature. After washing three times with PBS, they were incubated in 1:200 secondary antibody dilutions (Alexa Fluor 488-labeld goat anti-rabbit IgG [A-11008] and Alexa Fluor 568-labeled goat anti-mouse IgG [A-11004], Invitrogen, Carlsbad, CA, USA) for 1 h at room temperature. The membranes were washed and stained with 49,6-diamidino-2-phenylindole for 5 min at room temperature. Mowiol 4-88 (81381, Sigma-Aldrich) was used to mount the slides.

### 2.9. Outcome Measures

The clinical efficacy of the procedure was evaluated by comparing the following metrics preoperatively and postoperatively:

1. BCVA: BCVA was measured by the Tumbling E Chart.
2. Symblepharon grading: Grade 0: no symblepharon; Grade 1: limited to the conjunctiva; Grade 2: extending to the limbus; Grade 3: extending to the cornea [11].
3. Neovascularization grading: Grade 0: no neovascularization; Grade 1: confined to the limbus of the cornea; Grade 2: extending up to the margin of the pupil; Grade 3: extending beyond the margin of the pupil into the central cornea [11].
4. Conjunctivalization grading: Grade 0: no conjunctivalization; Grade 1: conjunctivalization involving less than one-quarter of the corneal surface; Grade 2: conjunctivalization involving one-quarter to one-half; Grade 3: conjunctivalization involving more than one-half of the corneal surface [11].
5. Cornea opacification grading: Grade 0: a clear cornea with clearly visible iris details; Grade 1: partial obscuration of the iris details; Grade 2: poor visibility of the iris details with a barely visible pupil margin; Grade 3: completely obscured iris and pupil details [11].
6. Fornix depth: The depth of the conjunctival fornix was measured in mm as the distance between the center of the superior and inferior fornix and corneal limbal in all participants. In healthy Asian populations as 8–10 mm for both superior and inferior fornices [12].

The primary outcome was defined as a completely epithelialized, absence of neovascularization and symblepharon invasion into the 5 mm diameter area of the central cornea. The secondary outcome for success was the improvement in BCVA by 2 lines or greater.

### 2.10. Statistical Analysis

Data are presented as mean ± SD. Due to the small sample size, normality tests were conducted on the data of conjunctival fornix depth. Wilcoxon paired test was used to compare preoperative and postoperative ocular surface grades and superior conjunctival fornix depth. Paired *t*-tests were used to compare the difference in inferior conjunctival fornix depth preoperatively and postoperatively. Statistical analysis was carried out using SPSS version 26.0 (SPSS, Inc., Chicago, IL, USA). A two-tailed *p*-value < 0.05 was considered statistically significant.

## 3. Results

### 3.1. Clinical Outcomes

**Figure 2:**
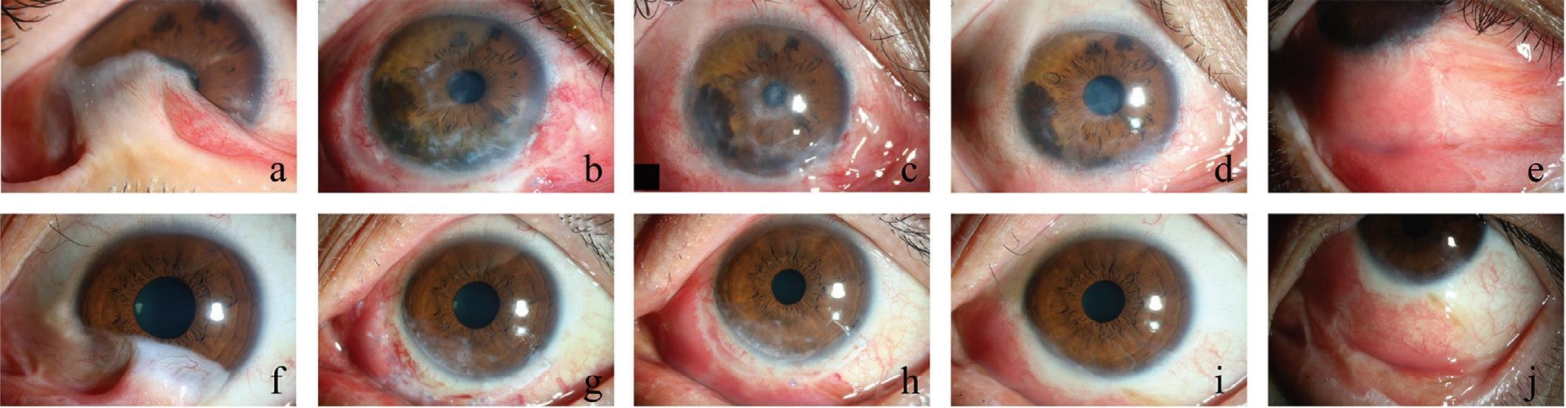
Clinical outcomes of Case 2 and Case 4. (a): Case 2 preoperative photograph. (b): After 1 month, the conjunctiva was congested, and the margins of KLAL segments were evident. (c): At 3 months, the corneal was epithelialized. (d and e): At 6 months, the cornea was clear, the color of the oral mucosa is consistent with the conjunctiva. (f): Case 4 preoperative photograph. (g): 1 week after surgery, segment and oral mucosal graft were in situ. (h): After 1-month, corneal opacification was reduced. (i and j): At 1 year, the ocular surface was smooth and clear, without recurrence of symblepharon.

In total 11 eyes of 10 patients were included in the study (nine males, one female; mean age, 41.70 ± 9.70 years old; range, 25–55 years old). All patients were graded as stage II–III LSCD, with alkali injury as the most common cause (50%). The mean duration of follow-up was 8.27 ± 5.80 months (3–22 months). At the final follow-up after surgery, primary successful outcomes were observed in 10 eyes (90.91%) of nine cases, that maintained a successfully regenerated stable corneal surface without persistent epithelial defects, progressive conjunctivalization, or vascularization. Postoperatively, statistically significant improvement was found in the grades of symblepharon (*Z* = −2.913, *p*=0.004), corneal vascularization (*Z* = −2.565, *p*=0.010), conjunctivalisation (*Z* = −2.850, *p*=0.004), and opacification (*Z* = −2.714, *p*=0.007). Superior conjunctival fornix depth increased from 5.09 ± 3.94 to 7.55 ± 1.57 (*Z* = −2.214, *p*=0.027). Inferior conjunctival fornix depth increased from 3.18 ± 3.52 to 7.64 ± 1.75 (t = −4.118, *p*=0.002). About eight eyes (72.73%) of seven cases showed significant improvement in BCVA. Recurrence of LSCD was observed in one recipient eye (9.09%). Clinical outcomes of Case 2 and Case 4 are shown in Figure 2.

### 3.2. In Vivo Confocal Microscopy

**Figure 3:**
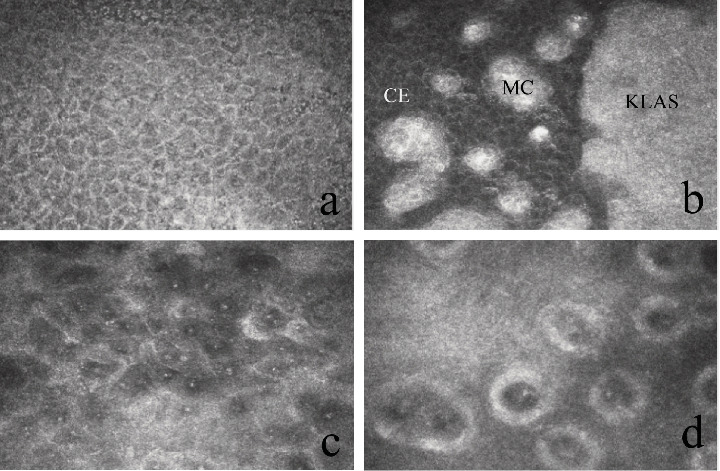
IVCM outcomes. (a, b): IVCM demonstrated multilayered corneal epithelial cells were intact without goblet cells, and the keratolimbal allograft segment was surrounded by mature epithelial cells. (c, d): IVCM showed conjunctival epithelial cells lining over the central cornea, and acinar-like structure was observed in the corneal limbus. IVCM images are 400 × 400 µm. KLAS: keratolimbal allograft segment; MC, mature cells; CE, cornea epithelium.

In successful cases, corneal epithelial cells were detected in the central cornea, without goblet cells (Figure 3a). Normal Vogt structure was not detected in the limbus. A large number of highly reflective mature epithelial cells surround the KLAL segments (Figure 3b). In the failed case, there were mixed corneal-conjunctival epithelial cells (mostly conjunctiva) lining over the central cornea (Figure 3c). The formation of acinar-like structures was observed in the corneal limbus (Figure 3d).

### 3.3. IC and IF

**Figure 4:**
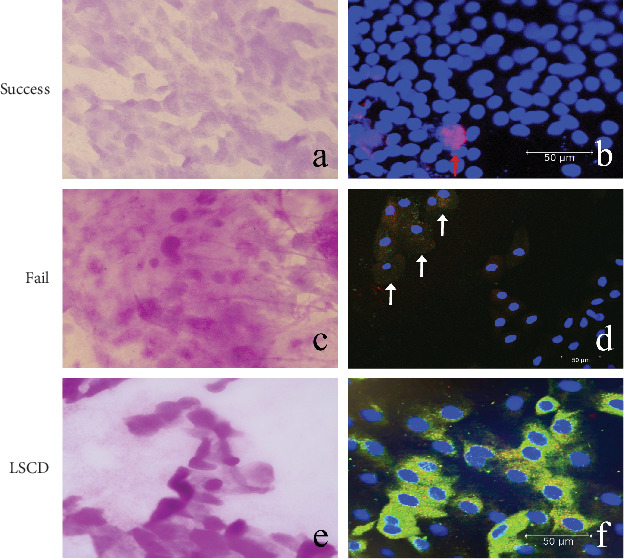
IC and IF outcomes. (a): IC showed a corneal phenotype without evidence of goblet cells. (b): P63-positive LSC (red arrow) was present in the corneal limbus. (c): IC revealed many strongly stained goblet cells covering the conjunctival epithelium cells. (d): MUC5AC- and CK7-positive goblet cells (white arrows) were distributed among the epithelial cells. (e): The central cornea was almost entirely composed of goblet cells. (f): MUC5AC- and CK7-positive goblet cells were distributed in the central cornea.

In patients with successful surgery, the corneal epithelial cells had clear borders, and the cells were polygonal and flat, without goblet cell infiltration (Figure 4a). IF showed that negative staining for Recombinant Mucin 5 Subtype AC (MUC5AC) and Recombinant Cytokeratin 7 (CK7). P63-positive LSC was found at the corneal limbus (Figure 4b). In patient with surgical failure, center of the cornea was replaced by conjunctival epithelium cells with blurred cell borders and strong PAS-stained goblet cells distributed in the epithelial cells (Figure 4c). MUC5AC- and CK7-positive goblet cells were distributed among the epithelial cells (Figure 4d). In patients with LSCD who did not receive surgery, the central cornea was composed of PAS-stained goblet cells (Figure 4e). IF showed the central cornea was distributed of a large number of MUC5AC- and CK7-positive goblet cells (Figure 4f).

## 4. Discussion

Reconstruction of the ocular surface in eyes with severe LSCD remains one of the most challenging problems in ophthalmology. It often leads to conjunctival ischemia, necrosis, and lysis of the eyelid, conjunctiva, and cornea, causing ocular surface scarring and symblepharon, disrupting normal ocular surface structures, and impairing visual acuity [13]. KLAL transplantation is an effective procedure for treating LSCD in which cadaveric LSCs are transplanted into the recipient's eye. The procedure was proposed by Thoft [14] in 1984. Innovative changes in surgical technique, preoperative management, and immunosuppression regimens have been developed in the last decade [15, 16]. However, whether partial or total LSCD, was performed using crescent-shaped implants. In this study, we used ring-shaped implants for patients with total LSCD, thereby simplifying the surgical steps and providing adequate LSCs because of the larger clock hours of available graft tissue.

To anatomically and functionally repair the eyelid, conjunctiva, and cornea, and to prevent the recurrence of symblepharon, we combined KLAL transplantation with OMT. The long-term outcomes of this surgery were reviewed, with a mean follow-up of 8.27 ± 5.80 months and in some cases up to 1–2 years. The results showed a 90.91% success rate for the procedure. The depth of the conjunctival fornix increased significantly in all patients. Only Case 5 experienced a recurrence of symblepharon due to the short period since the injury and the excessive immune-inflammatory response of the ocular surface. Previous studies showed that expression levels of inflammatory cytokines (IL-1β and IL-6), chemotactic cytokines (MCP-1 and MIP-1α), and TGF-β peaked within 1 week after ocular surface chemical burn and then gradually declined [17]. Therefore, burns >6 weeks were defined as the chronic phase [18]. However, the study by Kethiri et al. [19] concluded that the rabbit eye had complete conjunctivalization within 3 months after alkali burns and remained stable at 4 months. Therefore, we believe that surgical treatment should be performed after the ocular surface condition has stabilized for at least 1 month.

In addition to the recording of patient signs, the renewal of the corneal epithelium and the differentiation of LSCs were identified by IVCM, IC, and IF. Consistent with previous reports, both IVCM and IC showed multilayered corneal epithelia without conjunctival epithelial invasion in the central cornea was detected in successful cases [20–22]. The epithelial cells in the central cornea of the failed graft had lost the classic polygonal morphology [22–24]. Both IC and IF confirmed the infiltration of goblet cells in the central cornea. However, there were some conflicting results concerning the fate of transplanted LSCs in patients receiving KLAL transplantation. Hong et al. [20] found that 1 year after KLAL transplantation, Vogt's palisades extend into the limbal epithelium in a regular arrangement with blood vessels within. But our study found that the KLAL segments could be detected in the limbus, the limbus did not form Vogt's palisades, and there were a large number of cell islands formed by mature corneal epithelial cells around the grafts 1 year after surgery. We concluded that the donor KLAL segments could provide LSCs in the long term, which is consistent with the findings of Djalilian et al. [25] and Spelsberg et al. [26]. They compared the genotypes of surface epithelial cells of KLAL recipients with donor eyes and found that donor cells were detected 3.5 years postoperatively [25, 26].

To verify that KLAL grafts still have the potential to proliferate and differentiate in the long term, we performed IF on the limbus of the recipient's eye. P63 is a positive stem cell marker that is absent in the resting limbus [27]. In specimens with active proliferation, it was expressed in the upper basal layer of the corneal limbus, and it was hypothesized that the site of expression of this indicator would be stem cells and transiently expanding cells [28]. Clonal growth of p63-rich cell clusters was higher than in areas without clusters [29]. We found that P63-positive LSCs were present on the surface of the corneal limbus, suggesting that the grafts of the recipient eyes still have the potential to proliferate and differentiate 1 year after surgery.

KLAL transplantation with OMT is an innovative surgical approach for binocular LSCD or moderate to severe LSCD in one eye. Compared to AMT and conjunctival transplantation, it can reconstruct the anatomy of the conjunctival and eyelid, while providing a stable proliferative environment for the growth of LSCs [30]. This procedure improved the ocular surface homeostasis and patient's BCVA, with a higher surgical success rate and fewer complications compared to traditional KLAL transplantation. In addition, this was the first study to investigate the long-term limbal morphology after KLAL transplantation and found active proliferating LSCs on the limbal surface.

Limitations of this study, which was first reported from the Chinese population, include small sample size, short follow-up time for some patients. Therefore, compared with other large-sample, long-term follow-up studies, the surgical success rate is higher. In addition, this study only performed epithelial morphology analysis on five patients, which may have resulted in selection bias. Thus, more cases need to be accumulated, a longer observation period, and IVCM, IC, and IF before and after surgery to confirm the effectiveness of our technique.

To summarize, the preliminary results of this study suggest that KLAL transplantation with OMT may be a viable option for severe ocular surface diseases that require limbal reconstruction and conjunctival sac reconstruction to obtain a stable ocular surface. The surgical technique is easy to learn and safe to use.

## Figures and Tables

**Table 1:** Staging of LSCD.

Stage	Extent of corneal epithelial involvement	A	B	C
Stage I	Normal corneal epithelium within the central 5 mm zone of the cornea	<50% of limbal involvement	≥50% but <100% limbal involvement	100% of limbal involvement
Stage II	The central 5 mm zone of the cornea is affected	<50% of limbal involvement	≥50% but <100% limbal involvement	—
Stage III	The entire corneal surface is affected	—	—	—

**Table 2:** Demographic and clinical characteristics of the patients before surgery.

Case	Age (years old)	Sex	Eye	Etiology of LSCD (times)	Previous of surgery	Pre-op BCVA	Ocular surface appearance						Classifi-cation of LSCD
							Symbleph-aron (grade)	Vasculari-zation (grade)	Conjuncti-valization (grade)	Corneal Opacification (grade)	Fornix depth (mm)		
											Superior	Inferior	
1	55	M	OD	Alkali burn (4 months)	None	FC/BE	2	3	3	3	0	2	III
2	39	M	OD	Acid burn (5 months)	AMT	0.2	3	1	2	2	8	0	IIA
3	47	F	OD	Thermal burn (30 years)	AMT	FC/1m	3	3	2	2	8	0	IIA
			OS			0.02	3	2	2	2	5	6	IIB
4	29	M	OS	Thermal burn (17 months)	None	0.6	3	2	2	2	9	0	IIA
5	48	M	OD	Alkali burn (2 months)	Anterior chamber paracent-esis	HM	3	3	3	3	0	0	III
6	35	M	OS	Alkali burn (1 year)	AMT	0.01	1	3	1	2	9	4	III
7	32	M	OD	Thermal burn (7 months)	AMT	0.5	3	0	2	2	10	0	III
8	50	M	OS	Alkali burn (2 years)	AMT	HM	3	3	3	3	0	7	III
9	41	M	OD	Alkali burn (3 years)	None	0.1	3	3	2	2	4	7	IIB
10	25	M	OS	Alcohol burn (15 months)	None	FC/1m	3	3	3	3	3	9	IIB

Abbreviations: AMT, amniotic membrane transplantation; BCVA, best corrected visual acuity; BE, before eye; FC, figure count; HM, hand motion; LSCD, limbal stem cell deficiency; M, male; OD, right eye; OS, left eye; Pre-op, preoperative.

**Table 3:** Outcomes of patients who underwent surgery.

Case	Follow (months)	Post-op BCVA	Complications	Ocular surface appearance						Surgical results
				Symblepharon (grade)	Vascularization (grade)	Conjunctivalization (grade)	Corneal opacification (grade)	Fornix depth (mm)		
								Superior	Inferior	
1	4	0.3	None	0	2	0	1	7	8	S
2	6	0.5	None	0	1	0	0	8	9	S
3	12	0.1	None	0	1	0	2	8	7	S
	5	0.5	None	0	1	1	1	7	7	S
4	22	0.7	None	0	0	0	0	9	9	S
5	10	HM	Recurrent symblepharon	2	3	3	3	4	3	F
6	9	0.3	None	0	2	0	1	9	9	S
7	13	0.6	None	0	0	0	1	10	8	S
8	3	0.3	None	0	1	0	0	7	7	S
9	4	0.3	None	0	1	0	1	7	8	S
10	3	0.15	None	0	0	0	1	7	9	S

Abbreviations: BCVA, best corrected visual acuity; F, failure; HM, hand motion; Post-op, postoperative; S, success.

## Data Availability

The data that support the findings of this study are available upon request from the corresponding author. The data are not publicly available due to privacy or ethical restrictions.

## Nomenclature

LSCs: Corneal limbal stem cells
SJS: Stevens–Johnson syndrome
LSCD: Limbal stem cell deficiency
KLAL: Keratolimbal allograft
AMT: Amniotic membrane transplantation
OMT: Oral mucosal transplantation
BCVA: Best corrected visual acuity
IOP: Intraocular pressure
IVCM: In vivo confocal microscopy
IC: Impression cytology
IF: Immunofluorescence
HRT: Heidelberg retina tomograph
PAS: Periodic acid–Schiff
MUC5AC: Mucin 5 subtype AC
CK7: Recombinant cytokeratin 7.
